# Survival among treated tongue cancer patients: a single-center experience

**DOI:** 10.1007/s12672-024-00989-z

**Published:** 2024-04-23

**Authors:** Pablo Veiga-San Roman, Victor Villanueva San Vicente, M. Angeles Rodriguez-Gonzalez, Pia López-Jornet

**Affiliations:** 1 Cirugía Oral y Maxilofacial del Hospital Clínico Universitario Virgen de la Arrixaca Ctra., Madrid-Cartagena, s/n, 30120 El Palmar, Murcia Spain; 2 Pia López-Jornet Deparment Oral Medicine, University of Murcia Hospital Morales Meseguer, Clinica Odontologica Spain, Adv Marques de los Velez s/n, 30008 Murcia, Spain

**Keywords:** Survival, Tongue cancer, Oral squamous cell carcinoma, Oncological surgery

## Abstract

**Objective:**

To describe overall survival (OS) and disease-free survival (DFS) in a cohort of tongue cancer patients, together with the corresponding demographic, tumor and surgical characteristics.

**Methods:**

A retrospective study was made of 205 consecutive patients with primary tongue cancer subjected to surgery and adjuvant therapy according to the stage of the disease, in Hospital Clínico Universitario Virgen de la Arrixaca (HUVA) (Murcia, Spain) during the period 2000–2020. Survival was evaluated based on the Kaplan–Meier method, and the existence of significant differences between the different study variables was analyzed using the log-rank test. Cox regression analysis was performed for the identification of risk factors.

**Results:**

In relation to overall survival, 72.6% of the patients survived for a mean time of 14.43 years [standard error (SE) = 0.74; 95% CI: 12.98–15.87], with a cumulative survival rate of 49.8 ± 3%. Survival was reduced by the presence of tumor adjacent to resection margins [hazard ratio (HR) 2.20; 95% CI 1.09–4.43] (*p* = 0.028) and infiltrated resection margins (HR 3.86, 95% CI 1.56–9.57) (*p* = 0.004). Lymphadenectomy in turn increased survival (HR 0.15; 95% CI 0.06–0.42) (*p* < 0.001). In relation to disease-free survival, 55.3% of the patients suffered no relapse over a mean period of 9.91 years (SE = 0.66; 95% CI: 8.61–11.2), with a cumulative survival rate of 26.6% ± 8.4%.

**Conclusions:**

In tongue cancer patients, overall and specific survival were reduced in the presence of infiltrated resection margins. Lymphadenectomy in turn improved survival compared with patients in which this procedure was not carried out.

## Introduction

Oral squamous cell carcinoma (OSCC) is the most frequent neoplasm of head and neck cancer and accounts for 80–95% of those located in the oral cavity [1–4]. Excluding the lips, OSCC of the tongue is the most common oral cavity cancer (20–50%) [1–3].

The variability of the incidence of OSCC is related to the distribution of its multiple risk factors in the different geographical settings. Thus, in Asia and Southeast Asia, the incidence of OSCC of the tongue is very high and accounts for over one-third of the global world burden of OSCC [4].

Ng et al. [5] investigated the epidemiological changes of tongue cancer in a study involving over 80,000 patients in 22 countries. The authors found that the increase in incidence of this disease is indeed a worldwide phenomenon. Although the real population at risk is heterogeneous, there is clearly a tendency towards an increased frequency in younger patients [6]. An earlier age at presentation of the disease is associated to improved overall survival than in older patients, though the risk of local relapse is higher [7, 8]. These tendencies are a matter of concern and require improved understanding of the biology of these tumors, their genetic origins, and pathogenesis.

The treatment of choice is fundamentally surgical. Specifically, surgery is recommended in patients presenting early stage tumors, with surgery and concurrent chemotherapy–radiotherapy being indicated in advanced stages of the disease [6, 9].

There is important debate regarding neck treatment in early-stage OSCC (stages I and II), i.e., without cervical clinical manifestations. Both elective neck dissection and the “watchful waiting” strategy are advocated by different authors [10, 11]. The debate is to the fact that elective neck dissection adds needless costs and morbidity if no preoperative analysis is made to be 100% sure that there are no lymph node micrometastases at the time of the diagnosis. Failure to investigate this aspect will result in deficient regional control and a decrease in survival rate. Due to the high possibility of occult metastases in the lymph nodes of patients with clinically negative lymph node findings, the surgeon often performs high neck dissection. Although many prospective and retrospective studies have discussed the advantages and inconveniences of elective neck dissection, with different results, most authors consider that it offers benefits in terms of regional disease control and survival [10–19]. The present study analyzes the demographic, pathological and surgical characteristics of a retrospective cohort of patients with OSCC of the tongue treated in Hospital Clínico Universitario Virgen de la Arrixaca (HUVA)(Murcia, Spain), with the aim of determining the main factors conditioning the outcomes in terms of overall survival and disease-free survival.

## Material and methods

A retrospective observational study was carried out in the patients treated for OSCC of the tongue between 2000 and 2020 at the Department of Oral and Maxillofacial Surgery of Hospital Clínico Universitario Virgen de la Arrixaca (HUVA)(Murcia, Spain). The study was carried out in abidance with the principles of the Declaration of Helsinki, and was approved by the local Ethics Committee (Reference 2021-11-4-HUVA).

The inclusion criteria were patients with a histologically confirmed primary diagnosis of OSCC of the mobile tongue (only C02 site in ICD-O-3 was analyzed.), treated at the Department of Oral and Maxillofacial Surgery of HUVA. All the data were entered in a database designed in accordance with the good clinical practice guides. Patients with a prior history of cancer were excluded, as were individuals with other types of histological differentiation or tumor locations, and patients previously operated upon at some other hospital.

All the selected patients were treated using the United States guide on the treatment of OSCC as reference [20, 21]. This fundamentally involved surgical resection with safety margins, followed by elective or therapeutic cervical dissection (levels I–II–III). Adjuvant therapy in turn consisted of the following: patients with at least two risk factors (tumor size T4, infiltration of at least two lymph nodes, tumor adjacent to resection margins or depth of infiltration (DOI) > 10 mm) received radiotherapy, while those with extracapsular disease spread or tumor infiltration of resection margins received radiotherapy and adjuvant chemotherapy.

### Data collection

The study data were obtained retrospectively from the consecutive patient case histories. All the patients were diagnosed with OSCC of the mobile (free) tongue. Tumor staging was established from the histopathological biopsy report and the preoperative computed tomography (CT) study. Information regarding the prior surgical-medical history and preoperative laboratory tests (blood count, biochemistry, coagulation parameters) was collected for each patient. All the subjects were informed of the type of surgery to be carried out, and written consent was obtained in all cases. The multidisciplinary head and neck oncology committee of HUVA decided the management strategy in each case, based on the definitive pathology report. Data referred to mortality were recorded from the case history or from the Ágora database.

The following data were collected for each patient: age and gender, smoking habit (active smoker, ex-smoker, or never smoked), alcohol intake (regular consumption or no consumption), date of diagnosis, date of the pathology report confirming the diagnosis, tumor size (in mm, measured after tumor resection in the surgical piece and reflected in the pathology report), lymph node size and involvement (classified according to the NCCN guidelines version 2, 2023) [20], cervical lymphadenectomy (according to the surgical protocol employed, or no lymphadenectomy), cell differentiation and local infiltration (obtained from the pathology report on the resected tumor). With regard to postsurgical treatment, we recorded the need for adjuvant therapy, locoregional disease relapse during follow-up, and disease status at last patient revision. All the above were regarded as dependent variables, while the independent variables were defined as overall survival (time from surgery to death due to the neoplastic disease or any other cause.)

Disease specific survival is time from surgery to death only due to neoplastic disease (other causes are not recorded).disease-free survival is time from surgery to disease relapse or death due to any cause, what happens first.

### Statistical analysis

Basic descriptive statistics were recorded. Qualitative variables were reported as absolute numbers and percentages, while quantitative variables were reported as the mean and standard deviation (SD), maximum and minimum.

Kaplan–Meier survival analysis was used to explore differences in overall survival and disease-free survival in relation to the different demographic, tumor and surgical variables. In turn, Cox regression analysis was performed to determine the possible effect of the different demographic, tumor and surgical variables upon overall survival and disease-free survival; those variables that proved significant in the Kaplan–Meier analysis were entered in the model.

The SPSS version 27.0 statistical package was used throughout. Statistical significance was considered for *p* < 0.05.

## Results

The final study sample consisted of 205 patients, of which 62.4% were males (n = 128) and 37.6% females (n = 77). The mean age was 61.5 ± 14 years (range 25–91). About smoking habit, 60.8% of the patients were smokers (n = 121) and 39.2% were not (n = 78). Most of the patients were not regular drinkers of alcohol (57.5%; n = 113) while 42.3% were regular drinkers (n = 83).

Regarding tumor size (T) at the time of diagnosis, 112 patients presented a tumor size of ≤ 2 cm, 78 had a tumor size of > 2 cm or ≤ 4 cm, and 15 patients presented a tumor size of > 4 cm. The mean tumor size was 2 ± 1 cm (range 0.2–6).

In relation to lymph node involvement (pN), most of the patients presented no lymph node disease (n = 138) while 67 presented lymph node involvement, distributed as follows: 30 patients presented N1, 6 stage N2a, 23 stage N2b, one stage N2c, and 7 patients presented stage N3b. Ipsilateral lymphadenectomy was performed in 92% of the patients. A total of 147 underwent supra-omohyoid lymphadenectomy, 35 underwent functional cervical lymphadenectomy, 7 underwent radical cervical lymphadenectomy, and 19 patients were not subjected to lymphadenectomy. Contralateral cervical lymphadenectomy was performed in 7% of the patients. Regarding tumor cell infiltration of the lymph node capsule, 7% of the patients showed extracapsular disease spread. Lastly, the mean neutrophil–lymphocyte ratio (NLR) was 2.13 ± 1.3 (range 0.52–9.45).

The histological characteristics of the tongue tumors were distributed as follows: 42% were well differentiated OSCC, 55% were moderately differentiated OSCC, and 3% were undifferentiated OSCC. Most of the patients (78%) did not present perineural or lymphovascular invasion. Tumor budding (positive or negative) was recorded in 139 patients, and of these, 30% proved positive. The depth of infiltration (DOI) was recorded in 150 patients, and the mean value was found to be 8.5 ± 6 mm (range 1–45).

In relation to tumor stage, 63% of the patients presented early stage disease (stages I and II) at the time of diagnosis, while 37% of the patients presented advanced stages of the disease.

Twenty-seven percent of the patients received postoperative radiotherapy, 10% received radiotherapy and chemotherapy, and in 63% of the cases the postsurgery management protocol was limited to periodic reviews, since the tumors were in the early stages.

With regard to overall survival, 72.6% of the patients survived and 27.4% died. The mean overall survival was 14.43 years [standard error (SE) = 0.74; 95% CI: 12.98–15.87], with a cumulative survival rate of 49.8 ± 3%.

The demographic data and habits (Table 1) showed overall survival to be significantly lower among those patients over 45 years of age compared with younger individuals. With regard to the tumor characteristics survival was significantly poorer among the patients with infiltrated resection margins than in those with disease-free margins or tumor adjacent to resection margins (*p* = 0.003 and *p* < 0.001, respectively). In relation to the surgical variables (Table 1), survival was significantly poorer in the patients with extracapsular disease spread, lymphadenectomy and invasion than in the individuals without these findings.

**Table 1:** Overall survival according to demographic variables and habits. tumor, surgical characteristic variables

	Exitus		Survival % ± ET	Log rank	
	No	Yes		χ^2^(1)	*p*-value
Age				3.572	**0.047**
≤ 45 years	23 (85.2)	4 (14.8)	80.4 ± 9.6		
> 45 years	123 (70.7)	51 (29.3)	44.2 ± 13.3		
Smoker				3.477	**0.044**
No	62 (80.5)	15 (19.5)	48.4 ± 20.4		
Yes	81 (68.6)	37 (31.4)	57.7 ± 5.9		
Drinker				7.29	**0.007**
No	90 (80.4)	22 (19.6)	55.0 ± 16.5		
Yes	50 (62.5)	30 (37.5)	47.6 ± 7.7		
Margin status *n* (%)				17.673	** < 0.001**
Free > 5 mm	96 (82.1)	21 (17.9)	48.2 ± 20.1		
Next < 5 mm	40 (65.6)	21 (34.4)	57.3 ± 7.7		
Affected	6 (40)	9 (60)	25.7 ± 14.5		
Size, *n* (%)				1.229	0.268
T1/T2	136 (73.1)	50 (26.9)	50.5 ± 11.9		
T3/T4	10 (66.7)	5 (33.3)	48.1 ± 18.8		
Lymph node N0/N + , *n (%)*				9.85	**0.002**
N0	107 (78.1)	30 (21.9)	51.6 ± 15.4		
N +	39 (60.9)	25 (39.1)	47.6 ± 8.4		
Stage *n* (%)				10.292	**0.001**
Initial	102 (79.1)	27 (20.9)	52.8 ± 15.8		
Advanced	44 (61.1)	28 (38.9)	47.1 ± 7.9		
Neutrophil to lymphocyte ratio (NLR) (%), *media (DT)*	2.17 (1.37)	2.01 (1.11)			0.281
Extracapsular invasion				3.873	**0.047**
No	132 (74.6)	45 (25.4)	50.8 ± 12.1		
Yes	8 (61.5)	5 (38.5)	56.4 ± 15.2		
Lymphadenectomy				6.452	**0.011**
No	7 (43.8)	9 (56.2)	33.7 ± 14.8		
Yes	139 (75.1)	46 (24.9)	64.5 ± 4.9		
Invasion				4.77	**0.029**
No	112 (76.7)	34 (23.3)	64.5 ± 5.7		
Yes	26 (63.4)	15 (36.6)	57.2 ± 9.3		

The bold values are statistically significant.

Table 2 shows the results of the multivariate Cox regression analysis, carried out to determine the possible effect of the demographic, tumor and surgical variables upon survival. The infiltration of tumor resection margins had a significant impact upon overall survival, with decreased survival being observed among patients with infiltrated margins or tumor adjacent to resection margins versus those with disease-free resection margins. In turn, cervical lymphadenectomy resulted in significantly better survival than among the patients not subjected to lymphadenectomy.

**Table 2:** Cox regression. Effect of demographic, tumor and surgical variables on overall survival

	B (ET)	Wald	HR (IC 95%)	*p-*value
Age (≥ 45 vs. < 45)	0.63 (0.56)	1.29	1.88 (0.63–5.57)	0.256
Smoker (Yes vs. No)	0.31 (0.47)	0.43	1.36 (0.54–3.39)	0.512
Drinkers (Yes vs No)	0.07 (0.43)	0.03	1.07 (0.46–2.49)	0.874
Margin status				
Free > 5 mm	1			
Next < 5 mm	0.79 (0.36)	4.83	2.20 (1.09–4.43)	**0.028**
Affected	1.35 (0.46)	8.52	3.86 (1.56–9.57)	**0.004**
Size (T3T4 vs. T1T2)	0.27 (0.78)	0.12	1.31 (0.29–6.03)	0.729
N0/N + (N + vs. N0)	0.73 (0.84)	0.75	2.07 (0.40–10.73)	0.388
Stage (Advanced vs. Initial)	0.32 (0.86)	0.13	1.37 (0.25–7.44)	0.715
Extracapsular invasion (Yes vs. No)	−0.23 (0.57)	0.16	0.80 (0.26–2.45)	0.691
Lymphadenectomy (Yes vs. No)	−1.88 (0.52)	12.99	0.15 (0.06–0.42)	** < 0.001**
Invasion (Yes vs. No)	0.55 (0.40)	1.95	1.74 (0.80–3.78)	0.163

B, regression coefficient; ET, standard error; HR, Hazard ratio; CI, confidence interval

The bold values are statistically significant.

With regard to disease-free survival, 55.3% of the patients showed no disease relapse while 44.7% did experience disease relapse. The most severe decrease in disease-free survival was observed in the first two years, followed by a gradual decrease thereafter and during the entire remaining period of the study. The mean disease-free survival (i.e., time to relapse) was 9.91 years [standard error (SE) = 0.66; 95% CI: 8.61–11.2], with a cumulative survival rate of 26.6 ± 8.4%.

Table 3 present the data referred to demographic characteristics, habits, and tumor and survival variables. Disease-free survival was seen to be significantly lower among those patients over 45 years of age compared with younger individuals. Likewise, survival among the patients with disease-free resection margins was significantly greater than in those presenting margins with adjacent tumor or infiltrated margins (*p* = 0.046 and *p* < 0.001, respectively).

**Table 3:** Disease-free survival according to demographic, tumor characteristic,surgical variables

	Exitus		Survival % ± ET	Log rank	
	No	Yes		χ^2^(1)	*p*-value
Age				3.663	**0.046**
≤ 45 years	17 (63)	10 (37)	58.7 ± 12.1		
> 45 years	92 (54.1)	78 (45.9)	36.9 ± 5.7		
Smoker				0.232	0.63
No	44 (57.1)	33 (42.9)	42.2 ± 7.9		
Yes	63 (55.3)	51 (44.7)	38.2 ± 7.2		
Drinker				3.65	**0.048**
No	68 (61.3)	43 (38.7)	30.7 ± 11.9		
Yes	38 (49.4)	39 (50.6)	24.0 ± 8.3		
Margin status, *n (%)*				12.387	**0.002**
Free > 5 mm	73 (62.4)	44 (37.6)	34.2 ± 11.2		
Next < 5 mm	29 (50)	29 (50)	17.9 ± 13.5		
Affected	4 (26.7)	11 (73.3)	11.2 ± 10.1		
Tumor size, *n* (%)				0.242	0.623
T1/T2	100 (54.3)	84 (45.7)	25.8 ± 8.3		
T3/T4	9 (69.2)	4 (30.8)	61.5 ± 16.6		
N0/N + , *n* (%)				2.735	**0.048**
N0	78 (58.2)	56 (41.8)	28.5 ± 11.0		
N +	31 (49.2)	32 (50.8)	26.0 ± 8.2		
Stage, *n* (%)				1.516	0.218
Initial	73 (57.5)	54 (42.5)	28.0 ± 10.9		
Advanced	36 (51.4)	34 (48.6)	29.0 ± 8.1		
Neutrophil to lymphocyte ratio (NLR)	2.23 (1.42)	1.96 (1.11)			0.121
Extracapsular invasion				0.272	0.602
No	99 (56.9)	75 (43.1)	26.5 ± 10.2		
Yes	7 (58.3)	5 (41.7)	35.0 ± 25.8		
Lymphadenectomy				5.128	**0.024**
No	5 (33.3)	10 (66.7)	17.8 ± 14.2		
Yes	104 (57.1)	78 (42.9)	24.9 ± 9.7		
Invasion				1.979	0.159
No	84 (58.7)	59 (41.3)	33.9 ± 10.8		
Yes	20 (50)	20 (50)	34.7 ± 11.2		

The bold values are statistically significant.

Table 4 shows the results of the multivariate Cox regression analysis of the possible effect of the demographic, tumor and surgical variables upon disease-free survival. Infiltration of the surgical resection margins and the presence of tumor adjacent to the resection margins was seen to have a significant impact upon disease-free survival, with poorer patient survival than in the presence of disease-free resection margins. Likewise, the patients subjected to cervical lymphadenectomy showed better survival than those not subjected to lymphadenectomy.

**Table 4:** Cox regression. Effect of demographic, tumor and surgical variables on disease-free survival

	B (ET)	Wald	HR (IC 95%)	*p-*value
Age (≥ 45 vs. < 45)	0.56 (0.35)	2.59	1.76 (0.88–3.50)	0.108
Drinkers (Yes vs. No)	0.28 (0.24)	1.40	1.33 (0.83–2.13)	0.238
Margin status				
Free > 5 mm	1			
Next < 5 mm	0.44 (0.25)	3.01	1.55 (0.95–2.56)	**0.043**
Affected	0.96 (0.36)	7.13	2.62 (1.29–5.32)	**0.008**
N0/N + (N + vs. N0)	0.29 (0.26)	1.29	1.34 (0.81–2.23)	0.256
Lymphadenectomy	−0.89 (0.40)	5.08	0.41 (0.19–0.89)	**0.024**

B, regression coefficient; ET, standard error; HR, Hazard ratio; CI, confidence interval

The bold values are statistically significant.

## Discussion

Tongue cancer remains an aggressive disease due to its cellular biology and the absence of anatomical barriers against spread. Furthermore, this type of cancer presents a high risk of occult metastases in the early stages, with poor patient survival. In the present study, the multivariate Cox regression analysis found the presence of infiltrated surgical resection margins and the absence of cervical lymphadenectomy to be associated with poorer survival and greater disease relapse.

The incidence of OSCC of the tongue has increased in young individuals under 45 years of age, with no clear underlying etiological driving factor [6, 22–24]. Some authors such as Davidson et al. [22] have reported that survival is greater in younger individuals. They studied survival in a cohort of 749 patients divided into three age groups: < 40 years, ≥ 40 to ≤ 60 years, and > 60 years, and found the difference in survival between the first two groups and between the first and last group to be statistically significant (*p* < 0.05), though the difference between the two older groups failed to reach significance. Campbell et al. [23] likewise reported improved survival among younger individuals, though these were defined as patients under age 50.

In their systematic review, Lenze et al. [24] found the 5-year disease-free survival rate to range between 30% and 72% for the younger age groups and between 42–81% in the case of the older patients. There is still a lack of agreement as to whether or not the risk of disease relapse differs significantly with age. The growing incidence of the disease in younger and non-smoking individuals raises questions about the etiology, pathogenesis and prognosis of tongue cancer. In our study, the patients over 45 years of age did not exhibit epidemiological characteristics different from those of the older individuals [6, 24].

The neutrophil–lymphocyte ratio (NLR) is an inflammatory and immune response marker that is inexpensive and simple and easy to use, and is able to predict the patient clinical stage [25–27]. In this regard, Wang et al. [25] reported that NLR can be used to identify tongue cancer patients at an increased risk of occult cervical metastases. Wu et al. [26] in turn reported that NLR ≥ 2.95 inearly stage tongue cancer (cT1/T2N0) is associated to a more invasive tumor behavior. In contrast, Abbate et al. [27] found it difficult to establish a cut-off point for adequately guiding treatment choice.

The appropriate cervical treatment in early stage tumors (stages I and II), i.e., in patients with clinically negative neck findings, is subject to debate. Two large trials supported lymphadenectomy in patients with early-stage disease. D’Cruz et al. [10] conducted a single-center study involving 496 patients randomized to two treatment arms according to whether monitoring of the neck was decided, or the patients were subjected to elective lymph node dissection in the same surgical act as tumor removal. Of the studied patients, 85.3% presented OSCC of the tongue. Due to the high cervical disease relapse rate observed in the patients subjected to monitoring only, the trial had to be suspended on the basis of the criteria established by its protocol. The differences in overall survival and disease-free survival were 12.5 and 23.5 percentage points in favor of elective lymphadenectomy. In this context, regional lymph node relapse was the most frequent location of disease relapse.

Hutchinson et al. [11], in a multicenter study involving 596 patients with early stage OSCC, compared the possible benefits of elective dissection versus therapeutic lymphadenectomy (i.e., dissection performed once the tumor has relapsed). The authors evaluated overall survival, disease-free survival, locoregional relapse and adverse events after 6 months. In the two study groups (randomized to dissection versus observation), the differences in overall survival were not significant, while disease-free survival and locoregional relapse showed improved outcomes in the patients subjected to neck lymphadenectomy. In turn, adverse events were more frequent in the latter group of patients, though most of them were considered to be mild.

Infiltrated surgical resection margins worsen the prognosis, as seen in our study, with a decrease in overall and specific survival. Kurita et al. [28] also analyzed the risk of disease relapse according to the nearness of the tumor to the surgical resection margins. In the presence of adjacent tumor (≤ 5 mm from the resection margin), the risk of relapse was seen to increase 3.79-fold, versus 7.89-fold in the case of infiltration of the resection margins. After 5 years of follow-up, local disease control was 91% in the presence of disease-free margins, 80.4% in the presence of margins with adjacent tumor, 81.8% in the case of dysplasia of the surgical margins, and 43.8% in the presence of infiltrated margins.

In the meta-analysis carried out by Anderson et al. [29], the risk of relapse decreased by 21% if the disease-free resection margins measured more than 5 mm. In a retrospective cohort study of 539 patients, they found evidence that disease-free margins of less than 5 mm increased the risk of local disease relapse. Our own data are consistent with these observations.

Chang et al. [30] in turn analyzed the risk of relapse in 126 patients treated for OSCC of the tongue. Of these patients, 25 presented positive resection margins, which was associated to a 2.5-fold increase in the risk of local disease relapse. No association was observed between the surgical bed margin findings and the risk of local relapse. On analyzing the distance from the tumor to the resection margin, it was seen that the risk of relapse decreased 33% for every additional millimeter of separating distance.

The present retrospective study covers the period 2000–2020, and it must be noted that some of the histological descriptors were not analyzed on a routine basis during that entire period. In this regard, we were able to document the depth of infiltration (DOI) in 150 patients, and the presence of tumor budding (i.e., the presence of isolated or small clusters of tumor cells at the infiltrating front of the tumor) in 139 patients. In this respect, 30% of the patients presented budding, which was associated to a poor prognosis [31].

The strength of our study is the number of cases analyzed over a long follow-up period of 20 years. As limitations of the study, it was difficult to avoid selection bias, information bias or confounding effects in the context of our retrospective design, resulting in a lower level of evidence compared with other prospective studies.

Patient survival has changed little despite the technical improvements. We therefore need to explore those factors which can be controlled, in order to improve the patient prognosis, such as the time to diagnosis, treatment and hospitalization.

## Conclusions

The results of the present single-center study on squamous cell carcinoma of the mobile tongue show tumor infiltration of the surgical margins to reduce overall and specific survival. In turn, patients subjected to cervical lymphadenectomy have significantly better survival than patients not subjected to lymphadenectomy. Future prospective and multicenter studies are needed to further explore the factors that condition survival in these patients.

## Data Availability

The datasets used or analyzed during the current study are available from the corresponding authors on reasonable request.
